# Late Treatment and Recurrence of Kawasaki Disease in a Moroccan Infant

**DOI:** 10.1155/2019/3904932

**Published:** 2019-03-05

**Authors:** R. Elqadiry, O. Louachama, N. Rada, G. Draiss, M. Bouskraoui

**Affiliations:** Pediatric A Department, Mother-Child Pole, Mohammed VI University Hospital, Marrakesh, Morocco

## Abstract

**Introduction:**

While the diagnosis of typical form of Kawasaki disease (KD) is obvious, this multifaceted disease continues to surprise us. We report the case of a recurrent Kawasaki disease in an infant.

**Case:**

At the age of 13 months, the infant was diagnosed with complete Kawasaki disease; he presented with prolonged fever, bilateral conjunctivitis, enanthem, exanthema, edema of the lower limb, peeling, and biological inflammatory syndrome. He was treated with intravenous immunoglobulin (IVIG) associated with a high dose of aspirin and then an antiplatelet dose with a good clinical-biological evolution. The echocardiography was normal. Seven months later, the patient was again admitted, in a similar picture: a prolonged fever evolving for 7 days, bilateral conjunctivitis, enanthem, cervical adenopathy of 1.5 cm/1 cm, scarlatiniform erythema, pruriginous of the trunk and limb, and peeling of the toes, with indurated edema of the hands and feet. The rest of the examination was normal except the irritability. The diagnosis of recurrent KD was made according the five criteria of the American Heart Association. The echocardiography was normal again. The infant received IVIG with good outcome.

**Conclusion:**

Despite its rarity, the possibility of recurrence of KD should be known by clinicians, so as not to delay the specific management of vasculitis whose stakes in terms of prevention of coronary artery lesions are well known. Our case confirms the possibility of this recurrence.

## 1. Introduction

Kawasaki disease (KD) is an acute multisystemic vasculitis that affects young children and infants with predilection. After its first description in Japan, Kawasaki disease (KD) has been reported worldwide. Its incidence is variable from one country to another, and its severity was attributed from the outset, in the absence of diagnosis and treatment, to cardiovascular complications, mainly coronary.

The recurrence of KD is frequently reported in Japan and the USA, respectively, in 3-4% and 0.8% of cases [1], but it is rarely reported in Morocco.

We report a case of recurrent KD in its complete form and make through this observation a brief review of the literature.

## 2. Case

We report the case of a 20-month-old infant, whose parents are no consanguineous, the youngest of three siblings. Seven months ago, the diagnosis of complete form of KD was made because he presented with prolonged fever, bilateral conjunctivitis, enanthem, exanthema, edema of the lower limbs, and peelings and a biological inflammatory syndrome. The patient was treated with IVIG and acetylsalicylic acid with good outcome and no coronary abnormalities in echocardiography.

The infant was again admitted, 7 months later, in a similar picture: he had a fever at 39–40°C, persisting and resistant to the antipyretic drugs evolving for seven days, associated with a generalized scarlatiniform rash. The patient was initially treated in ambulatory with 3^rd^ generation cephalosporin and macrolide antibiotics without improvement and then referred to our department for further management.

**Figure 1:**
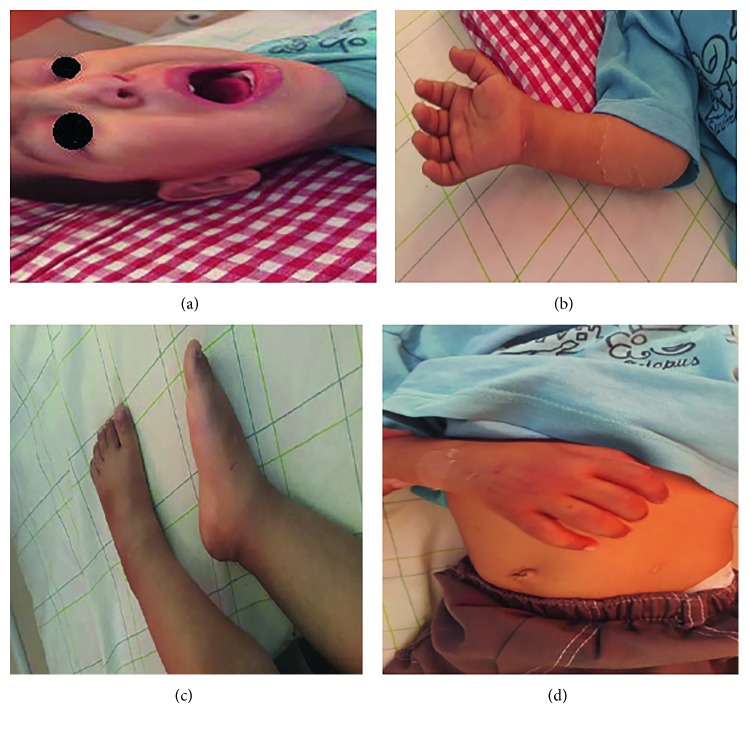
Clinical signs that made it possible to confirm KD.

Physical examination revealed an irritable infant, febrile with temperature at 39°C, icterus, bilateral nonpurulent conjunctivitis, bleeding cheilitis with “strawberry tongue,” scarlatiniform erythema, and pruriginous in the trunk and limbs associated with indurated edema of the hands and feet with peelings of the toes (Figure 1). Otherwise, examination of the lymph nodes noted noninflammatory cervical lymphadenopathy measuring 1.5 cm/1 cm.

Biological investigations showed an elevated leukocyte count with 20 100/mm^3^, with a predominance of neutrophils at 11,000/mm^3^ and thrombocytes at 7,61,000/mm^3^, elevated CRP at 104 mg/l, elevated SV at 86 mm at the first hour, and moderate elevations in serum transaminases (SGPT at 125 UI/l and SGOT at 80 UI/l). Urinanalysis revealed an aseptic leucocyturia, and the blood cultures were sterile.

The patient was treated with IVIG, 2 g/kg in a single infusion, together with high doses of aspirin (80 mg/kg/d) related by antiplatelet doses (3 mg/kg/d) after resolution of the inflammatory syndrome (in 4 weeks), according to the recommendations of the literature.

The infant has been afebrile after 48 hours of IVIG treatment, and the evolution was favorable, with regression of conjunctivitis and cutaneous signs and progression of CRP from 104 mg/l to 6 mg/l, and echocardiographic control was still normal.

## 3. Discussion

So many words express the many faces of Kawasaki. Since its first description in Japan, several hypotheses have been advanced, but no etiological factor has been identified.

The diagnosis of this lymphadeno-mucocutaneous syndrome, based on clinical criteria, can only be retained after excluding the other differential diagnosis [2].

Furthermore, the differential diagnosis of KD includes viral infections (measles, adenovirus, rubella, and mononucleosis) that present acute oropharyngitis, fever, and cervical lymphadenopathy, but with fewer systemic inflammatory signs and no involvement of the extremities. Of the same, the systemic juvenile idiopathic arthritis can mimic KD, but the absence of joint involvement after prolonged follow-up has excluded it in our patient. The patient had normal hemodynamic parameters, excluding streptococcal toxic shock. Furthermore, no improvement of symptomatology with 3^rd^ generation cephalosporin and macrolide excludes in our patient the possibility of scarlet fever and rickettsioses [3]. The diagnosis of recurrent KD was then retained and reinforced by the very high inflammatory syndrome. The good response of the two episodes to immunoglobulin infusion also reinforced our diagnosis.

The recurrence of KD is defined by the reappearance of symptoms two months after the first episode [4]. A Japanese study has tried to identify risk factors for this recurrence: age less than 2 years, prolonged fever over 10 days, anemia, high levels of transaminases, and coronary lesion during the first episode [5]. During the first episode, our patient had a prolonged fever because of the infusion delay of 18-day immunoglobulins. Outside of the young age, none of the other criteria were found. However, mucocutaneous jaundice was observed with cytolysis during the second episode.

The cases of KD recurrence are rare in Europe; on the contrary, the relapses were reported in Japan, China, Taiwan, Korea, and the United States at rates of 3%, 3.5%, 1.82%, 1.5%, and 0.8%, respectively. The disease appears to recur more frequently in the first two years after diagnosis, with the highest rates seen in children between 1 and 2 years of age, as in our patient [4].

## 4. Conclusion

The rarity of recurrence of KD and atypical clinical presentation made diagnosis difficult, but currently, any clinicians should know it. The major challenge of KD is diagnosing and treating it before irreversible coronary damage appears.

The recurrence of KD is possible, so the pediatricians should know it.
